# Site-Specific Response and Resistance Patterns in Patients with Advanced Non-Small-Cell Lung Cancer Treated with First-Line Systemic Therapy

**DOI:** 10.3390/cancers16112136

**Published:** 2024-06-04

**Authors:** Lauren Julia Brown, Julie Ahn, Bo Gao, Harriet Gee, Adnan Nagrial, Eric Hau, Inês Pires da Silva

**Affiliations:** 1 Department of Medical Oncology, Westmead Hospital, Sydney, NSW 2145, Australiaadnan.nagrial@sydney.edu.au (A.N.); ines.estevesdominguespiresdasilva@health.nsw.gov.au (I.P.d.S.); 2 Blacktown Cancer and Haematology Centre, Blacktown Hospital, Sydney, NSW 2148, Australia; 3 Faculty of Medicine and Health, University of Sydney, Camperdown, NSW 2050, Australia; 4 Westmead Institute for Medical Research, Westmead, NSW 2145, Australia; 5 Sydney West Radiation Oncology Network (SWRON), Sydney, NSW 2145, Australia; 6 Children’s Medical Research Institute, Westmead, NSW 2145, Australia; 7 Melanoma Institute Australia, Wollstonecraft, NSW 2065, Australia

**Keywords:** NSCLC, immunotherapy, chemoimmunotherapy, bone metastases, liver metastases, brain metastases, radiotherapy

## Abstract

**Simple Summary:**

Immunotherapy or combined chemoimmunotherapy is currently first-line therapy for patients with metastatic NSCLC without a driver mutation. Despite immunotherapy contributing to improved survival outcomes, the estimated 5-year OS rate for metastatic NSCLC remains poor. The aim of our retrospective study was to assess how patients with different anatomical metastatic sites respond or develop resistance to immunotherapy, combination chemoimmunotherapy, or chemotherapy alone. We confirmed that patients with bone metastases have poorer survival outcomes compared to those without bone metastases. This highlights a group of patients that may benefit from a specific clinical trial evaluation to assess the benefit of additional novel therapeutics or upfront radiotherapy.

**Abstract:**

Patients with advanced NSCLC have heterogenous responses to immune checkpoint inhibitors (ICIs) with or without chemotherapy. In NSCLC, the impact of the distribution of metastatic sites and the response to systemic therapy combinations remain poorly understood. In a retrospective cohort study of patients with unresectable stage III/IV NSCLC who received first-line systemic therapy, we sought to assess the association between the site of metastases with patterns of response and progression. Data regarding demographics, tumour characteristics (including site, size, and volume of metastases), treatment, and outcomes were examined at two cancer care centres. The endpoints included organ site-specific response rate, objective response rate (ORR), progression-free survival (PFS), and overall survival (OS). Two-hundred and eighty-five patients were included in the analysis. In a multivariate analysis, patients with bone metastases had a reduced ORR, PFS, and OS. Primary resistance was also more likely in patients with bone metastases. Patients with bone or liver metastases had a shorter OS when receiving ICIs with or without chemotherapy, but not with chemotherapy alone, suggesting an immunological basis for therapeutic resistance. A directed assessment of the tumour microenvironment in these locations and a deeper understanding of the drivers of organ-specific resistance to immunotherapy are critical to optimise novel combination therapies and sequencing in these patients.

## 1. Introduction

Lung cancer remains the leading cause of cancer-related death worldwide [1]. Non-small-cell lung cancer (NSCLC) is the most common subtype of lung cancer. The advent of immunotherapy has led to a significant improvement in outcomes for patients with metastatic NSCLC. Immune checkpoint inhibitors (ICIs) targeting programmed cell death receptor 1 (PD-1) and its ligand (PD-L1) including pembrolizumab, atezolizumab, and nivolumab are now the standard of care for the first-line management of patients with metastatic NSCLC with or without chemotherapy. In the first-line, ICIs alone or in combination with chemotherapy have also been shown to improve progression-free survival (PFS) and overall survival (OS) in patients with metastatic NSCLC without a driver mutation [2,3,4,5]. Additionally, combination immunotherapy with ipilimumab and nivolumab has been shown to be superior to chemotherapy alone for the management of metastatic NSCLC [6]. The efficacy of anti-PD-(L)1 inhibitors has also now been established in early stage, resectable [7,8,9,10,11,12], and non-resectable stage III NSCLC [13]. Despite immunotherapy contributing to improved survival outcomes, the estimated 5-year OS rate for metastatic NSCLC remains very poor—between 10 and 20% [1].

The patterns of metastatic spread in NSCLC are well described, based on registry data [14], retrospective analyses [15,16], and autopsy studies [17]. The most common extrathoracic metastatic sites for patients with NSCLC are the liver, brain, bone, and adrenal glands [14,15,16,17]. However, there is significant heterogeneity between studies with regards to the reported frequencies of metastatic involvement, depending on the histologic subtype. This is of significance as retrospective analyses have suggested that metastases to different organs may have differential responses. For example, liver metastases are less responsive to immunotherapy, whilst lymph node metastases have a higher response rate to ICIs in both metastatic NSCLC [18,19] and melanoma [20,21,22].

Exactly how the response to systemic therapy, including ICI and chemotherapy combinations in the first-line, is influenced by the distribution of metastatic involvement remains poorly understood. The incremental benefit of chemotherapy in particular metastatic sites remains unknown. We sought to assess the association between the site of metastases with patterns of response, progression, and survival in a cohort of patients with advanced non-small-cell lung cancer receiving first-line immunotherapy, chemoimmunotherapy, or chemotherapy.

## 2. Materials and Methods

### 2.1. Patients and Treatment

We retrospectively identified two-hundred and eighty-five consecutive patients who were treated at two tertiary cancer centres in NSW, Australia between January 2014 and September 2021 (allowing for all patients to have at least 24 months of follow-up). Patients were included if they received first-line systemic therapy for the management of advanced NSCLC (defined as metastatic, stage IV NSCLC, or stage IIIB/C NSCLC untreatable with definitive chemoradiation). Patients were treated with either first-line (1) immunotherapy: anti-PD-1 antibody alone (nivolumab, pembrolizumab or atezolizumab), or combination anti-CTLA-4 (ipilimumab) and anti-PD-1 (nivolumab); (2) chemoimmunotherapy: histology-driven platinum doublet chemotherapy in combination with an anti-PD-1 antibody (nivolumab or pembrolizumab); or (3) chemotherapy alone. As per international guidelines, patients who received immunotherapy alone had a PD-L1 expression of ≥50%. Patients received chemotherapy in addition to immunotherapy if they had a PD-L1 expression of <50% or if it was deemed appropriate by the treating clinician. Patients were treated with chemotherapy alone prior to 2018 when anti-PD-1 antibodies became available via government reimbursement in Australia or in cases where patients had pre-existing conditions contraindicating immunotherapy.

Approval from the Institutional Ethics Review Board (Western Sydney Local Health District Human Research Ethics Committee; 2020/ETH02064) was obtained. In accordance with the approved protocol, the requirement for obtaining individual patient consent was waived.

The data collected included patient demographics, tumour histology, location, volume and size of metastatic disease, treatment details including response rates at different metastatic sites, progression details, and survival outcomes. The volume of disease was defined as low, <5 metastases; moderate, 5–20 metastatic lesions; and high, >20 metastases. Extracranial lesions measuring ≥ 5 mm and cranial lesions measuring ≥ 2 mm were included in the analysis of volume of disease. The number of metastatic deposits measuring ≥ 5 cm was recorded.

### 2.2. Disease Assessment

Computed tomography (CT) of the chest, abdomen, and pelvis (3-mm slices) was obtained at baseline (before starting treatment), then every 12–16 weeks or more frequently, according to institutional practice. All patients had imaging of the brain prior to commencement of systemic therapy, either with CT or magnetic resonance imaging (MRI). In patients with known brain metastases, they were followed up with brain MRI every 12–16 weeks.

In each patient, metastatic lesions were measured on CT or MRI before and during treatment if they were ≥5 mm in the long axis, brain metastases ≥ 2 mm in the long axis, and lymph node [LN] metastases ≥ 15 mm in the short axis. Site-specific response in individual organs was recorded for all patients. This was classified as a complete response (CR) (disappearance or reduction to <10 mm in the short axis for an LN metastasis), a partial response (PR) (≥30% reduction), stable disease (SD) (neither a CR, PR, nor progressive disease [PD]), or PD (≥20% growth) in the affected organ. For bone metastases, this was assessed as either SD or PD given most bone lesions without a soft tissue component do not achieve a PR or CR. For patients with brain metastases, or other lesions, that had treatment with prior local therapy (radiotherapy, SRS or surgery) were assessed as achieving SD or PD. If other assessable cranial lesions had not been treated with local therapy, then the intracranial site-specific response could be assessed as per the other sites. For patients receiving immunotherapy (either alone or in combination with chemotherapy), the baseline volume of disease was recorded for each patient by organ site and assessment of the percentage change in disease volume was recorded for all lesions at each time point.

Early progression (primary resistance) was defined as progression within 6 months of commencement of systemic treatment, whereas late progression (acquired resistance) was defined as PD after an initial response (PR or CR) or SD for at least 6 months.

### 2.3. Statistical Assessment

Descriptive analysis was used for baseline characteristics. Continuous variables were summarised using medians and interquartile ranges (IQRs). Categorical variables were summarised using proportions. A Chi-squared test was employed to compare categorical variables between two independent groups. A nonparametric test (Mann–Whitney U test) was used to assess continuous variables between two independent groups. Objective response rate (ORR) was defined as the proportion of patients who had a partial response (PR) or complete response (CR) to treatment. OS and PFS were calculated from the date of commencement of treatment to the date of an event (either death [OS] or progression [PFS]). Patients without a clinical event were censored at the last follow-up date. Univariate and multivariate analyses of ORR were conducted using logistic regression modelling. Univariate and multivariate time-to-event analyses (OS and PFS) were conducted using the Cox regression analysis. Predictors of response, OS, and PFS, used in the models, included tumour and patient characteristics, size, volume and location of metastases, and treatment type. All statistical analyses were performed using R (version 4.2.3, R Foundation for Statistical Computing, Vienna, Austria) and some graphics were created using GraphPad Prism (version 10.0.2).

## 3. Results

### 3.1. Patient Characteristics

Two-hundred and eighty-five patients with advanced NSCLC were included. Patients received first-line treatment with either single-agent immunotherapy, chemotherapy or combination of chemoimmunotherapy. The median age was 68 years (IQR 60, 74), 174 (61%) were male, 74 (26%) were current smokers and 170 (60%) had a past history of smoking. Two-hundred and forty-four (86%) had an ECOG status of 0–1. The histological subtypes of NSCLC included 181 (64%) with adenocarcinoma, 67 (24%) with squamous cell carcinoma, 29 (10%) with undifferentiated large-cell carcinoma and 8 (3%) with not otherwise specified (NOS) carcinoma. Of the patients who had PD-L1 testing on their tumour, 58 (58 of 211, 27%) were PD-L1 negative, 52 (52 of 211, 25%) were PD-L1 1–49%, and 101 (101 of 211, 48%) were PD-L1 ≥ 50%. Seventy-four (26%) patients did not have routine PDL1 testing, given that this was not present in routine clinical practice before 2017 (Table 1). Twenty-two (7.7%) patients had stage IIIC NSCLC, unable to be treated with surgery or definitive chemoradiation, and 263 (93%) had stage IV disease.

One-hundred and six (37%) patients received chemoimmunotherapy, 96 (34%) received immunotherapy (7 of which received combination ipilimumab and nivolumab) and 83 (29%) received chemotherapy alone. Radiotherapy prior to first-line systemic therapy was received by 98 patients (39%).

Sites of metastatic disease before starting systemic therapy included 151 (53%) with lung metastases (separate to the primary lung lesion), 236 (83%) with lymph node metastases; either thoracic or extra-thoracic, 95 (33%) with pleural disease, 85 (30%) with bone, 60 (21%) with brain, 53 (19%) with adrenal and 47 (16%) with liver metastases.

### 3.2. Objective Response Rate

**Figure 1:**
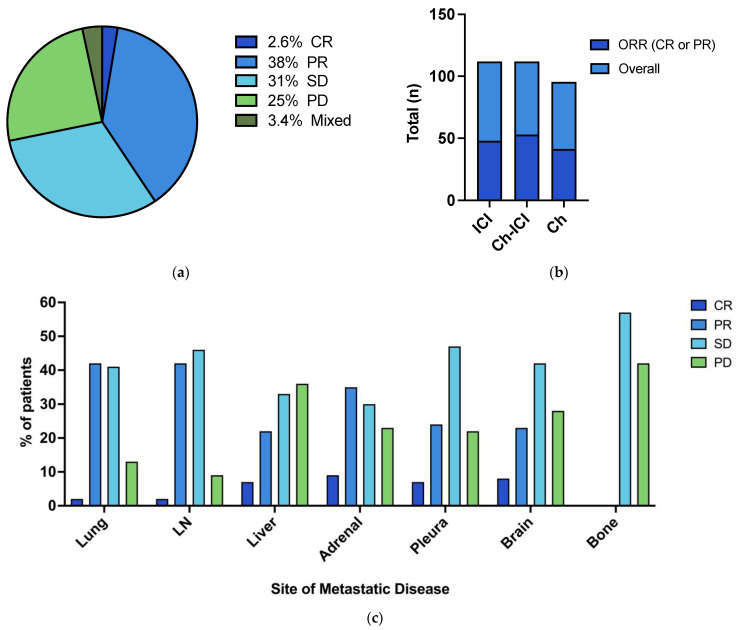
(**a**) Objective response rate in entire cohort; (**b**) Objective response rate by systemic therapy; (**c**) Site-specific response rate. Legend: CR: complete response; PR: partial response; SD: stable disease; PD: progressive disease; ICI: Immune checkpoint inhibitor; Ch: Chemotherapy.

With a median follow-up time of 45 months (IQR 23; 71 months), the ORR was 41%. Of the 266 patients with a recorded response, 7 (2.6%) had CR recorded as their best response, 101 (38%) had PR, 83 (31%) had SD, 66 (25%) had PD and 9 (3.4%) had mixed response (Figure 1a).

In total, 37 (13%) patients in the cohort had a response for ≥24 months including 22 treated with immunotherapy alone, 9 treated with chemoimmunotherapy, and 6 with chemotherapy.

The ORR was 44% (47 of 106) for patients who received chemoimmunotherapy, 30% (32 of 96) for those who received immunotherapy, and 27% (29 of 83) in patients treated with chemotherapy (*p* = 0.4) (Figure 1b). Multivariate analysis including all covariates and treatment type confirmed the presence of bone metastases was associated with a reduced likelihood of achieving a response (OR 0.35; 95% CI 0.16–0.73; *p* < 0.01). Pleural disease (OR 1.93; 95% CI 1.02–3.74; *p* = 0.05) and history of smoking (OR 6.67; 95% CI 2.09–23.3; *p* < 0.01) were associated with an increased likelihood of response (Table 2, Supplementary Table S1).

### 3.3. Site-Specific Response Rate

The patterns of response were also evaluated at each site of disease, measured as the best response at each site or anatomical location (Figure 1c). Lung lesions (43.9%), adrenal (44.2%), and lymph node metastases (43.5%) had the highest proportion of lesions achieving a complete or partial response compared with the other sites of disease. In contrast, bone (41.7%), liver (35.6%), and brain metastases (28.3%) had a higher proportion of progressive disease as their best response compared with the other sites of disease.

**Figure 2:**
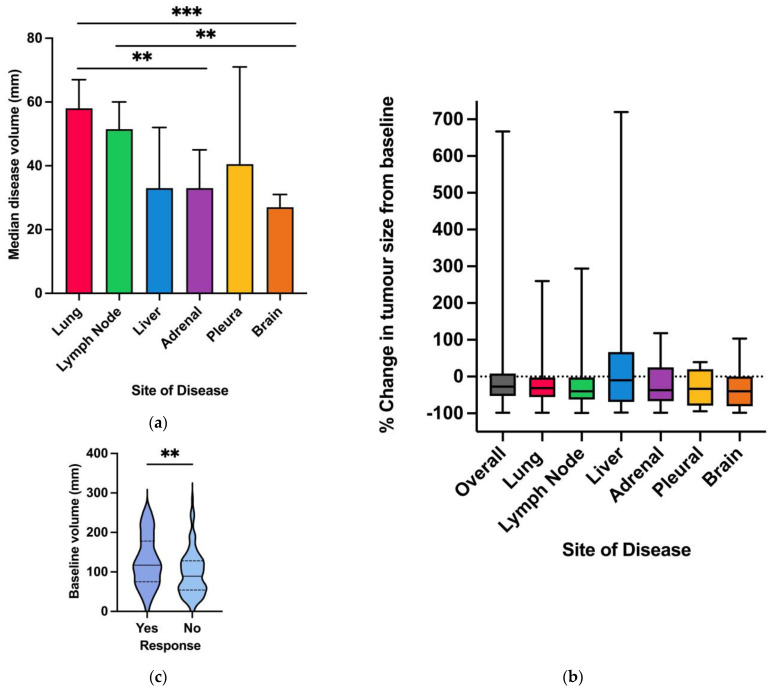
(**a**) Baseline median tumour volume in entire cohort; (**b**) Percentage change in tumour volume by site in immunotherapy and chemoimmunotherapy cohort; (**c**) Baseline tumour volume and objective response rate in immunotherapy and chemoimmunotherapy cohort; legend: **: *p* < 0.01; ***: *p* < 0.001.

The median disease volume at baseline at each site was largest in the lung (58 mm) and smallest in the brain (27 mm) (Figure 2a). In the cohorts of patients who received ICIs (immunotherapy and chemoimmunotherapy cohorts), the best change in tumour volume from baseline varied according to the site of disease; the greatest regression was observed in the lymph nodes (median −40%; range −100% to 260%), brain (median −40%; range −100% to 103%), adrenal glands (−37%; range −100% to 117%), lung (median −27%; range −100% to 260%), and pleura (median −33%; range −100% to 40%). Liver metastases had the lowest tumour volume regression (median −10%; range −100% to 720%) (Figure 2b). Patients with a higher burden of disease at baseline were more likely to have an objective response (median volume 127 mm vs. 97 mm; *p* < 0.001) (Figure 2c).

Of the 60 patients with brain metastases, 40 underwent local intracranial therapy prior to systemic therapy. Additionally, 17 (17 of 60, 28%) patients had surgical resection followed by cavity stereotactic radiosurgery (SRS), 12 (12 of 60, 20%) received SRS alone, 10 (10 of 60, 17%) received whole brain radiotherapy (WBRT), and one (1.7%) received surgery alone. Of the patients who had local therapy alone, 4 of 20 had an intracranial response (20%), with 10 patients having stable disease (50%). Of the 17 (17 of 60, 28.3%) patients who had progressive disease as their best response, 6 received local therapy alone, 6 underwent resection and cavity SRS, 3 received SRS alone, 1 received WBRT, and 1 received surgery alone.

### 3.4. Progression-Free Survival

**Figure 3:**
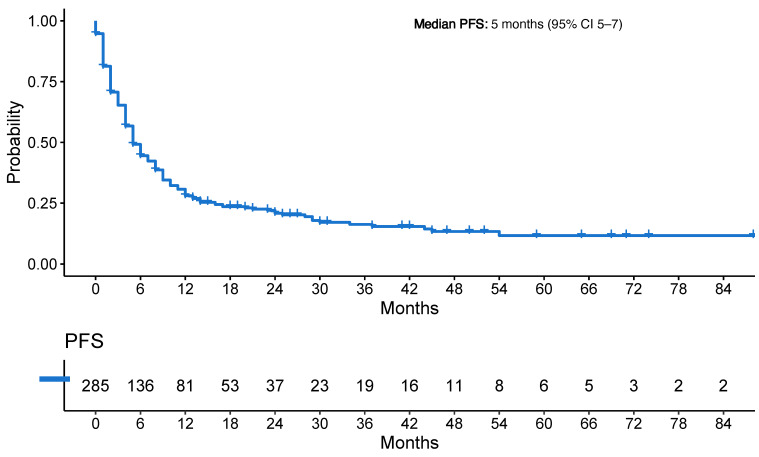

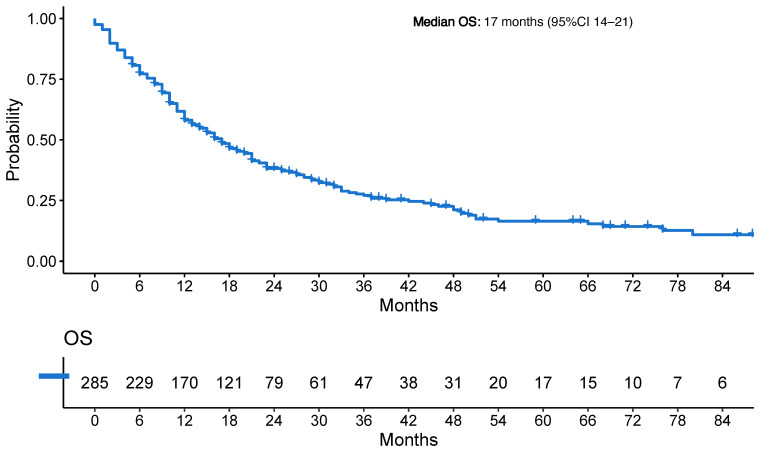
(**a**) Progression-free survival of entire cohort; (**b**) Overall survival of entire cohort. Legend: PFS: progression-free survival; OS: overall survival; CI: confidence interval.

The median PFS was 5 months (95% CI 5–7 months) (Figure 3a). The median PFS was 4 months on chemotherapy (95% CI 4–5 months), 6 months on immunotherapy (95% CI 4–10 months), and 8 months on chemoimmunotherapy (95% CI 6–12 months; *p* < 0.001) (Supplementary Figure S1).

**Figure 4:**
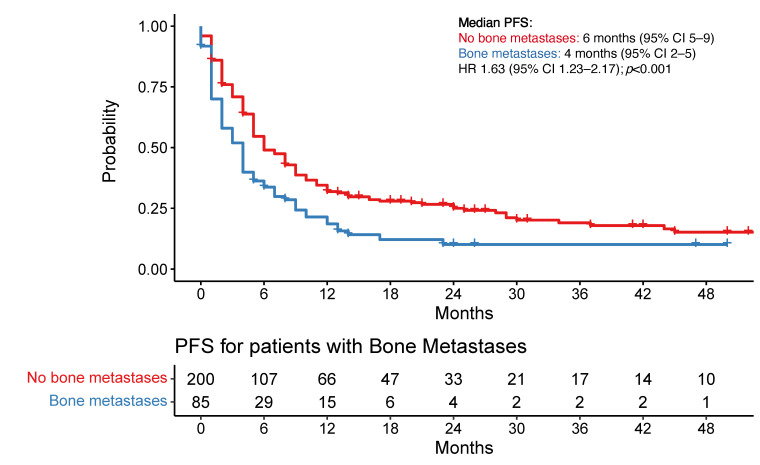

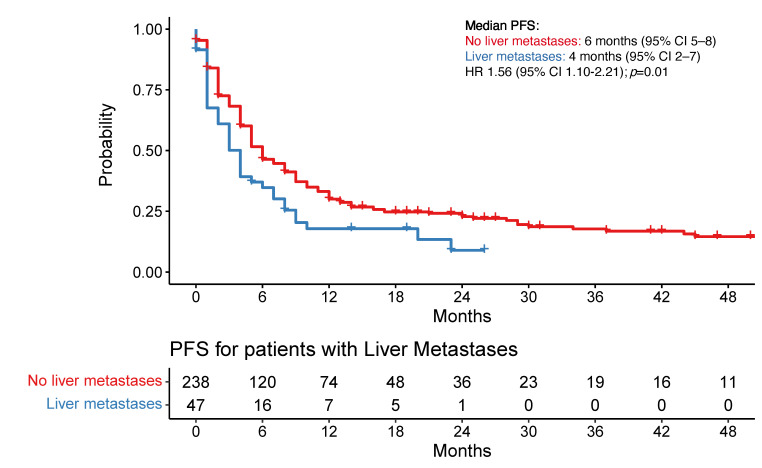

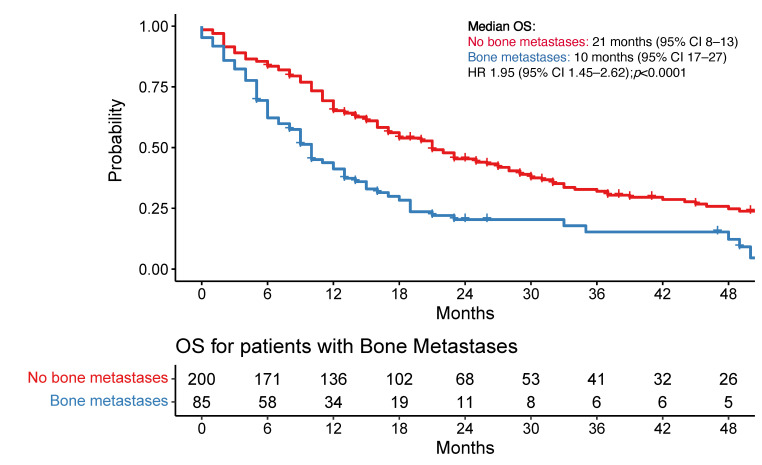

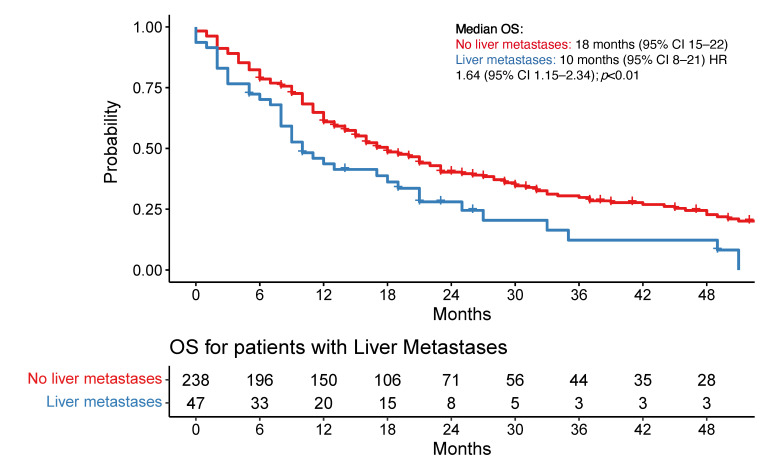
(**a**) Progression-free survival by bone metastases; (**b**) Progression-free survival by liver metastases; (**c**) Overall survival by bone metastases; (**d**) Overall survival by liver metastases. Legend: PFS: progression-free survival; OS: overall survival; CI: confidence interval; HR: hazard ratio.

Patients with bone metastases had a median PFS of 4 months (95% CI 2–5 months). By contrast, PFS was 6 months (95% CI 5–9 months) in those without bone metastases (HR 1.63 (95% CI 1.23–2.17); *p* < 0.001) (Figure 4a). Patients with liver metastases had a median PFS of 4 months (95% CI 2–7 months) versus 6 months (95% CI 5–8 months) in those without liver metastases (HR 1.56 (95% CI 1.10–2.21); *p* = 0.01) (Figure 4b).

Multivariate analysis confirmed the presence of bone metastases (HR 1.70; *p* < 0.01) and presence of a metastatic lesion ≥ 5 cm at any anatomical site (HR 1.74; *p* < 0.01) were associated with shorter PFS. Patients treated with immunotherapy with (HR 0.34) or without chemotherapy (HR 0.52) was associated with a longer PFS (*p* < 0.01). PD-L1 positive disease (PDL1 ≥ 1% HR 0.74, PDL1 ≥ 50% HR 0.63; *p* < 0.01) and history of smoking (Ex-smoker HR 0.51, current smoker HR 0.61; *p* = 0.02) were associated with improved PFS (Table 2, Supplementary Table S2). No difference was noted in PFS for patients who received upfront radiotherapy.

### 3.5. Overall Survival

The median OS for all patients was 17 months (95% CI 14–21 months) (Figure 3b). The median OS was 19 months (95% CI 16–28 months) for patients on chemotherapy, 15 months (95% CI 11–22 months) for patients on immunotherapy, and 16 months (95% CI 12–23 months) on chemoimmunotherapy (*p* = 0.72) (Supplementary Figure S2).

Patients with bone metastases had a median OS of 10 months (95% CI 8–13 months) versus 21 months (95% CI 17–27 months) in those without bone metastases (HR 1.95 (95% CI 1.45–2.62); *p* < 0.0001) (Figure 4c). Patients with liver metastases had a median OS of 10 months (95% CI 8–21 months) versus 18 months (95% CI 15–22 months) in those without liver metastases (HR 1.64 (95% CI 1.15–2.34); *p* < 0.01) (Figure 4d). There was no statistically significant difference in OS or PFS for other sites of metastatic disease.

The multivariate analysis confirmed that the presence of bone metastases (HR 2.01; *p* < 0.001), a high volume of disease (HR 1.66; *p* = 0.03), and squamous histology (HR 2.05; *p* < 0.01) were associated with shorter OS. (Table 2, Supplementary Table S3). No difference was noted in OS for patients who received upfront radiotherapy.

Patients with bone metastases (Supplementary Table S4) or liver metastases (Supplementary Table S5) had a shorter OS when receiving chemoimmunotherapy or immunotherapy compared to patients without bone and liver metastases. There was no difference in the OS of patients with bone metastases vs. no bone metastases or liver metastases vs. no liver metastases receiving chemotherapy.

### 3.6. Primary and Acquired Resistance

Progression occurred in 227 patients. Of these, 147 (65%) developed early (primary) resistance and eighty (35%) developed acquired resistance. Early (primary) resistance was more common in patients who received chemotherapy (69%) and acquired resistance was more common in patients who received chemoimmunotherapy (40%).

Patients with bone disease were more likely to experience early progression compared to those without bone lesions (76.1% vs. 59.6%, *p* = 0.02), whereas patients with pleural disease were more likely to experience late progression or acquired resistance compared to those without pleural disease (41.3% vs. 30.6%; *p* = 0.03). There was no difference in the timing of progression for those with liver, brain, or adrenal disease.

## 4. Discussion

For patients with metastatic NSCLC, response patterns to ICIs are variable and so too are the survival outcomes. Small studies have demonstrated that the distribution of metastatic sites predicts the response to immunotherapy in NSCLC [18,19,21,23]. We present the first data examining the influence of metastatic site on important clinical outcomes in patients receiving first-line chemoimmunotherapy vs. immunotherapy vs. chemotherapy alone, as well as the influence of radiotherapy. Our results highlight the important influence of the metastatic distribution on therapeutic response, in particular immunotherapy response. In our study, we have demonstrated the following: (1) Patients with bone metastases had a reduced ORR, PFS, and OS; (2) patients with bone or liver metastases have a shorter OS when receiving ICIs with or without chemotherapy; (3) patients with bone metastases were more likely to experience primary resistance.

Despite 20–48% of patients with metastatic NSCLC having bone metastases at diagnosis [24,25,26], there are limited clinical trials that specifically report on the outcomes of this subpopulation. This may be partly due to the focus on liver or brain metastases [27,28], which are reported more commonly in clinical trials, owing to known poorer prognostic implications [6,29,30,31,32,33,34,35]. Our results confirm previous findings from the Checkmate 9LA study of first-line nivolumab, ipilimumab, and chemotherapy compared with chemotherapy, which demonstrated poorer OS in patients receiving the chemoimmunotherapy combination for patients with bone metastases [34]. Those with bone metastases had a median OS of 11.9 months (HR 0.74; 95% CI 0.53–1.01) compared with those without bone metastases of 20.5 months (HR 0.65; 95% CI 0.51–0.82). The Checkmate 227 study of first-line nivolumab plus ipilimumab versus chemotherapy also reported similar results [6]. Patients with bone metastases receiving dual immunotherapy had a median OS of 13.4 months (HR 0.75; 95% CI 0.55–1.03) vs. 18.8 months (HR 0.81; 95% CI 0.67–0.99) in those without bone metastases. A large retrospective analysis of patients with NSCLC who received immunotherapy also demonstrated reduced immunotherapy efficacy and OS in patients with bone metastases [36].

The reduced ORR, OS, and PFS in patients with bone metastases suggests there is a systemic impact from the presence of bone metastases. There are several factors contributing to the potential immune resistance and decreased efficacy of immunotherapy in patients with bone metastases. Known as the “bone marrow niche”, this specialised microenvironment safeguards disseminated tumour cells from immune surveillance [37]. The complex interplay among immune cells, myeloid cells, and stromal cells within the bone yields both anti-tumour and tumour-promoting effects. While certain immune cells can trigger anti-tumour responses, others, such as myeloid-derived suppressor cells [38], regulatory T cells [39], and tumour-associated macrophages [40], contribute to immune suppression and facilitate tumour growth. This intricate dynamic creates an immunosuppressive environment, potentially leading to immunotherapy resistance within the bone. Prior retrospective studies investigating bone modifying agents such as bisphosphates or denosumab in addition to immunotherapy are yet to demonstrate any difference in survival outcomes for these patients [41,42,43]. A single-arm study of second-line nivolumab combined with denosumab demonstrated the efficacy of this combination [44]. However, randomised trials of chemotherapy ± denosumab have not shown any additional survival benefit [45,46]. Given that the basis of resistance in the bone appears to be immune related, further prospective evaluation specifically evaluating the benefit of first-line ICIs ± bone modification agents or denosumab in patients with NSCLC and bone metastases would be beneficial.

We also observed that patients with liver metastases exhibited a lower PFS and OS in comparison to patients without liver metastases. However, this difference did not reach statistical significance in the multivariate analysis. The presence of liver metastases is a poor prognostic factor for patients with lung cancer [27]. Prior studies of melanoma have demonstrated reduced ORR, PFS, or OS to either anti-PD-1 monotherapy [20,21] or combination immunotherapy [22] in patients with liver metastases. This pattern of response and survival outcomes in patients with liver metastases has also been demonstrated in NSCLC [21,47] and other cancers including hepatocellular carcinoma [48], microsatellite stable colorectal cancer [49], and triple-negative breast cancer [50,51] treated with anti-PD1 monotherapy. A previous study conducted on melanoma patients showed a distinct change in the systemic cytokine profile among patients with liver metastases compared to those without liver metastases [52]. In this study, reduced responses were also observed in the extra-hepatic metastases, suggesting that the existence of liver metastases might induce a systemic immunosuppressive effect leading to poorer survival outcomes. Furthermore, a recent assessment of the liver tumour microenvironment in NSCLC has demonstrated lower proportions of CD8 T cells within the liver compared with paired primary lung samples [53]. This study also demonstrated antigen processing pathways, natural killer cell cytotoxicity, and T-cell receptor signalling pathways were reduced in liver tumour samples compared to the primary lung tumour, providing a basis for immunotherapy resistance in patients with NSCLC and liver metastases. A deeper understanding of immunosuppressive mechanisms in liver metastases are required to overcome resistance.

Our study did not reveal improved outcomes for patients with bone or liver metastases who received chemoimmunotherapy when compared to those who received immunotherapy alone. This is yet to be evaluated in prospective trials. Notably, a real-world analysis involving PDL1-high NSCLC patients treated with either chemoimmunotherapy or immunotherapy alone exhibited no differences in outcomes for individuals with liver metastases [54].

While our study cohort did not include patients treated with both chemoimmunotherapy and VEGF inhibitors, post-hoc analyses from the IMpower-150 trial involving NSCLC patients with liver metastases have indicated that those who received a combination of carboplatin, paclitaxel, atezolizumab, and bevacizumab experienced improved outcomes compared to those who received only bevacizumab and chemotherapy [32]. A recent meta-analysis highlighted that the addition of VEGF inhibition has demonstrated the potential to enhance the response to immunotherapy in liver metastases, both in NSCLC and other cancers [55]. This introduces a potential treatment avenue for patients with liver metastases that requires further prospective evaluation.

Patients with a higher burden of disease at baseline were more likely to have an objective response (*p* < 0.001). This is contrary to what has previously been demonstrated in melanoma, where smaller metastases were more likely to achieve a CR than larger metastases [22]. Whilst patients with a higher burden of disease were more likely to have achieved an objective response, our study demonstrated that those with ≥20 metastases were associated with a lower OS (HR 1.66, *p* = 0.03). This confirms the findings of a prior study demonstrating that higher tumour volume was associated with poorer OS outcomes in metastatic NSCLC treated with anti-PD1 therapy [56].

Patients with bone metastases have a higher rate of primary resistance than those who did not. Improving our understanding of which patient phenotypes have primary immunotherapy resistance should direct clinical trial development targeting these resistant populations. The use of stereotactic body radiotherapy (SABR) prior to pembrolizumab in patients with metastatic NSCLC has demonstrated improved PFS and OS in the PD-L1-negative subgroup [57]. This suggests the role of upfront radiotherapy may be of benefit in patients with primary resistance to immunotherapy. Although our study did not confirm this, this may be due to the retrospective nature of the study, dose and location of radiotherapy or sequencing of radiotherapy. The SABR-COMET trial assessed the use of SABR for patients with oligometastatic disease, to all metastatic sites in combination with standard-of-care systemic therapy, with the aim of cytoreduction. This demonstrated an improvement in OS for patients with oligometastatic disease [58,59], with 35% of patients receiving SABR to bone metastases and 13% to liver metastases. Hypofractionated radiotherapy in a pooled analysis of two phase II trials has also demonstrated the ability to re-invigorate immunotherapy responses in patients with resistance to ICIs with NSCLC, leading to ongoing disease control [60].

Several novel therapies may also help to overcome resistance mechanisms in resistant organ sites. Preclinical NSCLC studies have demonstrated that the synergistic use of anti-PD-1 therapy in addition to antibody–drug conjugates improves response in immunotherapy-resistant models [61]. Numerous trials are currently underway assessing antibody drug conjugates in combination with immunotherapy including EVOKE-02 (NCT05186974), TROPION-Lung02 (NCT04526691) and -04 (NCT04612751), an AXL-ADC with PD-1 inhibitor (NCT04681131). However, further data are awaited regarding the efficacy of these combinations in specific organ sites and resistant patient populations. Given the success of radionuclide therapies for the management of castration-resistant prostate cancer with extensive bone metastases [62,63,64], further efforts exploring this option in managing bone metastases in other solid organ tumours are required.

### Limitations

There are several limitations of our study. Firstly, its retrospective design introduces the potential for selection bias, such as inadvertently excluding patients lost to follow-up. Secondly, the study is confined to two cancer centres and involves a small sample size. Consequently, the groups were heterogeneous, particularly the chemotherapy group, which was smaller and had fewer patients with bone metastases. This variability may have influenced the identification of predictive factors associated with outcomes and survival. Finally, not all patients in this study had baseline PET imaging or WBBS imaging to assess the presence of bone metastases, an issue that is broad across prospective clinical trials, with the use of bone scans usually only mandated if the patient is symptomatic. Thus, there is a risk that small metastases at some sites may not have been detected and included in the volume of metastases assessment. As bone metastases are not always accurately assessed using CT imaging [65,66], it is also possible that patients who had asymptomatic bone metastases were not included in the bone metastases cohort, thereby reducing the accuracy of the conclusions made. Additionally, patients with bone metastases were only assessed as SD or PD due to the difficulty with measurement of bone disease, which may have limited the accuracy of the site-specific response assessment in the bone. 

## 5. Conclusions

This detailed analysis of response patterns to systemic therapy in NSCLC suggests that treatment outcomes vary depending on the metastatic site of disease. This is the first study to analyse site-specific response patterns in NSCLC receiving chemoimmunotherapy. Poor outcomes were especially significant in patients with bone and liver metastases undergoing chemoimmunotherapy and immunotherapy, suggesting that there may be underlying immunosuppressive factors within these organs contributing to resistance and reduced survival. Further investigation is needed to deepen our understanding of the specific tumour microenvironment in these locations and the drivers of organ-specific resistance to immunotherapy. This is critical to determine the optimal treatment type and sequencing for patients with bone and liver metastases with NSCLC and to improve treatment response and outcomes.

## Figures and Tables

**Table 1:** Baseline Characteristics.

Characteristic	All n = 285 (%)	Immunotherapy n = 96 (%)	Chemoimmunotherapy n = 106 (%)	Chemotherapy n = 83 (%)
Age *	68 (60, 74)	70 (62, 75)	66 (58, 72)	67 (60, 73)
**Sex**				
Female	111 (39)	36 (38)	40 (38)	35 (42)
Male	174 (61)	60 (63)	66 (62)	48 (58)
**ECOG PS**				
0	105 (37)	27 (28)	41 (39)	37 (45)
1	139 (49)	52 (54)	56 (53)	31 (37)
≥2	41 (14)	17 (18)	9 (8)	15 (18)
**Tumour Type**				
Adenocarcinoma	181 (64)	54 (56)	68 (64)	59 (71)
SCC	67 (24)	28 (29)	22 (21)	17 (20)
ULCC	29 (10)	12 (13)	12 (11)	5 (6)
NOS	8 (3)	2 (2)	4 (4)	2 (2)
**PD-L1 Expression**				
Tested	211 (74)	81 (84)	102 (96)	28 (34)
<1%	58 (27)	2 (2.4)	40 (39)	16 (57)
≥1%	52 (25)	9 (11)	33 (32)	10 (36)
≥50%	101 (48)	70 (83)	29 (28)	2 (7.1)
Not tested	74 (26)	15 (16)	4 (3.8)	55 (66)
**Upfront RT**				
Radiotherapy	98 (34)	37 (39)	46 (43)	15 (18)
No radiotherapy	187 (66)	59 (61)	60 (57)	68 (82)
**Lung Metastases**				
Not present	134 (47)	47 (49)	55 (52)	32 (39)
Present	151 (53)	49 (51)	51 (48)	51 (61)
**LN Metastases**				
Not present	49 (17)	16 (17)	21 (20)	12 (14)
Present	236 (83)	80 (83)	85 (80)	71 (86)
**Brain Metastases**				
Not present	225 (79)	76 (79)	81 (76)	68 (82)
Present	60 (21)	20 (21)	25 (24)	15 (18)
**Liver Metastases**				
Not present	238 (84)	86 (90)	84 (79)	68 (82)
Present	47 (16)	10 (10)	22 (21)	15 (18)
**Bone Metastases**				
Not present	200 (70)	71 (74)	62 (58)	67 (81)
Present	85 (30)	25 (26)	44 (42)	16 (19)
**Adrenal Metastases**				
Not present	232 (81)	76 (79)	83 (78)	73 (88)
Present	53 (19)	20 (21)	23 (22)	10 (12)
**Pleural Metastases**				
Not present	190 (67)	64 (67)	73 (69)	53 (64)
Present	95 (33)	32 (33)	33 (31)	30 (36)

* Interquartile range. Legend: n: number; ECOG PS: Eastern Cooperative Oncology Group Performance Status; SCC: squamous cell carcinoma; ULCC: undifferentiated large-cell carcinoma; NOS: not otherwise specified; PD-L1: programmed cell death ligand 1 receptor; RT: radiotherapy.

**Table 2:** Significant Associations with Response and Survival in the Multivariate Analysis.

	ORR		PFS		OS	
**Characteristic**	OR (95% CI)	*p*-Value	HR (95% CI)	*p*-Value	HR (95% CI)	*p*-Value
**Pleural Metastases**		0.05				
Not present	1.00					
Present	1.93 (1.02, 3.74)					
**Bone Metastases**		<0.01		<0.01		<0.001
Not present	1.00		1.00		1.00	
Present	0.35 (0.16, 0.73)		1.70 (1.17, 2.47)		2.01 (1.37, 2.97)	
**Smoking Status**		<0.01		0.02		
Never Smoked	1.00		1.00			
Ex-Smoker	3.06 (1.11, 9.19)		0.51 (0.32, 0.82)			
Current Smoker	6.67 (2.09, 23.3)		0.61 (0.35, 1.06)			
**Treatment Type**				<0.001		
Chemotherapy			1.00			
Immunotherapy			0.52 (0.31, 0.86)			
Chemoimmunotherapy			0.34 (0.21, 0.56)			
**Metastatic lesion ≥ 5 cm**				<0.01		
Not present			1.00			
Present			1.74 (1.21, 2.50)			
**PD-L1 Status**				0.01		
<1%			1.00			
≥1%			0.74 (0.44, 1.24)			
≥50%			0.63 (0.41, 0.97)			
**Tumour Type**						<0.01
Adenocarcinoma					1.00	
SCC					2.05 (1.39, 3.04)	
ULCC					0.88 (0.47, 1.63)	
NOS					0.36 (0.05, 2.74)	
**Volume of metastases**						0.03
<20 metastases					1.00	
≥20 metastases					1.66 (1.05, 2.62)	

Legend: ORR: objective response rate; PFS: progression-free survival; OS: overall survival; OR: odds ratio; HR: hazard ratio; CI: confidence interval; PD-L1: Programmed Cell Death Ligand 1; SCC: squamous cell carcinoma; ULCC: undifferentiated large-cell carcinoma; NOS: not otherwise specified.

## Data Availability

The data for this study is unavailable due to privacy or ethical restrictions.

## Supplementary Materials

The following supporting information can be downloaded at: https://www.mdpi.com/article/10.3390/cancers16112136/s1, Figure S1: PFS by treatment type; Figure S2: OS by treatment type, Table S1: Univariate and Multivariate analysis for ORR; Table S2: Univariate and Multivariate analysis for PFS; Table S3: Univariate and Multivariate analysis for OS; Table S4: OS and PFS for bone metastases by treatment type; Table S5: OS and PFS for liver metastases by treatment type.
