# Supplementary material for: Site-Specific Response and Resistance Patterns in Patients with Advanced Non-Small-Cell Lung Cancer Treated with First-Line Systemic Therapy
Source: Cancers (Basel). 2024 Jun 4;16(11):2136. doi: 10.3390/cancers16112136 (PMC11172392; doi:10.3390/cancers16112136)
Supplement: Supplementary file 1 [file cancers-16-02136-s001.zip › cancers-3011980-supplementary.pdf]

Supplementary Material

Supplementary Figures:

Supplementary Figure S1: PFS by treatment type
Supplementary Figure S2: OS by treatment type

Supplementary Tables:

Supplementary Table S1: Univariate and Multivariate analysis for ORR
Supplementary Table S2: Univariate and Multivariate analysis for PFS
Supplementary Table S3: Univariate and Multivariate analysis for OS
Supplementary Table S4: OS and PFS for bone metastases by treatment type
Supplementary Table S5: OS and PFS for liver metastases by treatment type

Brown, L.J.; Ahn, J.; Gao, B.; Gee, H.; Nagrial, A.; Hau, E.; Silva, I.P. Site-Specific Response and Resistance Patterns in Patients with Advanced Non-Small-Cell Lung Cancer Treated with First-Line Systemic Therapy. *Cancers* **2024**

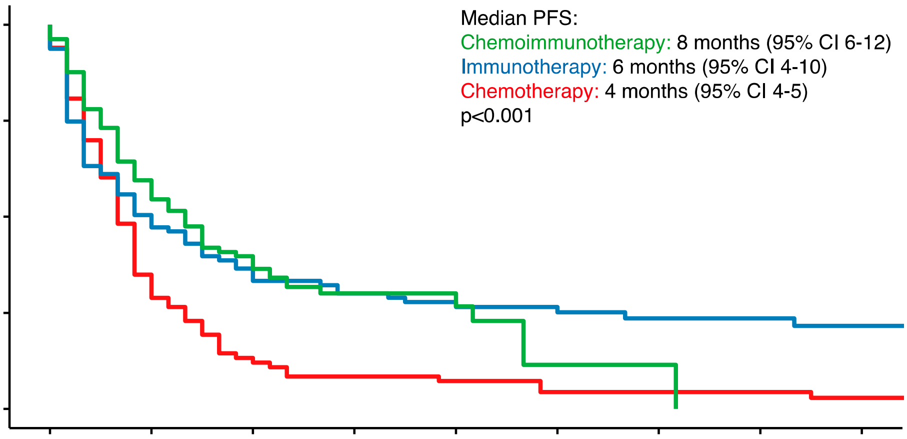

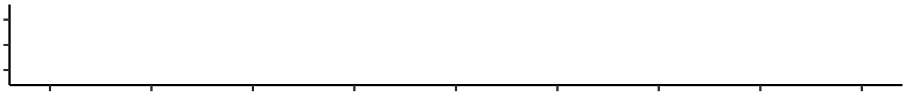

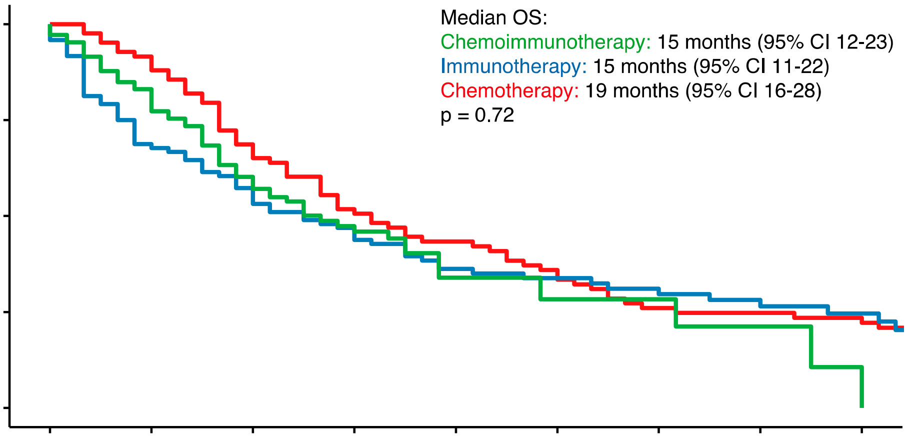

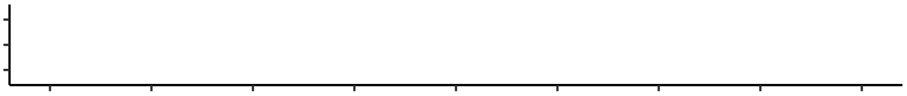

Legend: : PFS: Progression Free Survival; OS: Overall Survival; CI: Confidence Interval

Brown, L.J.; Ahn, J.; Gao, B.; Gee, H.; Nagrial, A.; Hau, E.; Silva, I.P. Site-Specific Response and Resistance Patterns in Patients with Advanced Non-Small-Cell Lung Cancer Treated with First-Line Systemic Therapy. *Cancers* **2024**

Supplementary Table S1: Univariate and Multivariate analysis for ORR

	Univariate			Multivariate		
**Characteristic**	**OR[1]**	**95% CI[1]**	**p-value**	**OR[1]**	**95% CI[1]**	**p-value**
**Lung Metastases**			0.20			0.40
Not present	1.00	—		1.00	—	
Present	1.38	0.85, 2.27		1.28	0.68, 2.44	
**Lymph Node Metastases**			0.53			0.20
Not present	1.00	—		1.00	—	
Present	0.82	0.43, 1.55		0.57	0.26, 1.25	
**Liver Metastases**			0.32			0.30
Not present	1.00	—		1.00	—	
Present	1.40	0.72, 2.73		0.66	0.28, 1.52	
**Brain Metastases**			0.25			0.14
Not present	1.00	—		1.00	—	
Present	0.70	0.37, 1.28		1.95	0.81, 4.79	
**Adrenal Metastases**			0.97			0.60
Not present	1.00	—		1.00	—	
Present	0.99	0.53, 1.82		0.83	0.39, 1.73	
**Pleural Metastases**			0.06			**0.05**
Not present	1.00	—		1.00	—	
Present	1.66	0.99, 2.79		1.93	1.01, 3.74	
**Bone Metastases**			**<0.001**			**<0.01**
Not present	**1.00**	**—**		**1.00**	**—**	
Present	**0.37**	**0.20, 0.66**		**0.35**	**0.16, 0.73**	
**Volume of metastases**			0.68			0.70
<20 metastases	1.00	—		1.00	—	
≥20 metastases	0.87	0.44, 1.68		1.16	0.48, 2.74	
**Metastatic lesion ≥5cm**			0.08			0.20
Not present	1.00	—		1.00	—	
Present	1.63	0.94, 2.82		1.55	0.79, 3.03	
**Age**			0.38			0.4
<65 years	1.00	—		1.00	—	
≥65 years	1.01	0.99, 1.04		1.01	0.98, 1.05	
**Sex**			0.75			>0.9
Female	1.00	—		1.00	—	
Male	1.08	0.66, 1.80		1.01	0.54, 1.90	
**Smoking Status**			0.13			**<0.01**
Never-Smoker	1.00	—		1.00	—	
Ex-Smoker	1.63	0.77, 3.64		**3.06**	**1.11, 9.19**	
Current Smoker	2.32	1.02, 5.55		**6.67**	**2.09, 23.3**	
**PD-L1 Status**			0.54			0.90
<1%	1.00	—		1.00	—	
≥1%	0.96	0.43, 2.15		1.06	0.39, 2.84	
≥50%	1.51	0.76, 3.01		1.14	0.49, 2.67	
Not Tested	1.17	0.56, 2.43		0.82	0.31, 2.16	
**Tumour Type**			0.80			0.80
Adenocarcinoma	1.00	—		1.00	—	
SCC	1.04	0.57, 1.88		0.74	0.34, 1.58	

ULCC	0.74	0.31, 1.65		0.64	0.23, 1.71	
NOS	0.35	0.02, 2.43		0.49	0.02, 6.58	
**Treatment Type**			0.38			0.20
Chemotherapy	1.00	—		1.00	—	
Immunotherapy	0.87	0.46, 1.65		0.48	0.17, 1.36	
Chemoimmunotherapy	1.30	0.71, 2.40		1.61	0.58, 4.59	
**Radiotherapy**			0.13			0.60
No Upfront RT	1.00	—		1.00	—	
Upfront RT	0.66	0.38, 1.12		0.84	0.42, 1.66	

Legend: n: number; SCC: Squamous Cell Carcinoma; ULCC: Undifferentiated Large Cell Carcinoma; NOS: Not otherwise specified; PD-L1: Programmed Cell death ligand 1 receptor; RT: Radiotherapy

Supplementary Table S2: Univariate and Multivariate analysis for PFS

	Univariate			Multivariate		
**Characteristic**	HR[1]	95% CI[1]	p-value	HR[1]	95% CI[1]	p-value
**Lung Metastases**			0.88			0.70
Not present	1.00	—		1.00	—	
Present	1.02	0.79, 1.33		0.94	0.67, 1.33	
**Lymph Node Metastases**			0.19			0.60
Not present	1.00	—		1.00	—	
Present	1.27	0.88, 1.82		0.88	0.58, 1.34	
**Liver Metastases**			0.74			0.50
Not present	1.00	—		1.00	—	
Present	0.95	0.68, 1.31		1.16	0.75, 1.80	
**Brain Metastases**			**0.02**			0.60
Not present	**1.00**	**—**		1.00	—	
Present	**1.56**	**1.10, 2.21**		1.13	0.72, 1.77	
**Adrenal Metastases**			0.20			0.60
Not present	1.00	—		1.00	—	
Present	0.80	0.56, 1.14		0.88	0.58, 1.34	
**Pleural Metastases**			0.60			0.30
Not present	1.00	—		1.00	—	
Present	1.08	0.82, 1.41		1.21	0.86, 1.69	
**Bone Metastases**			**0.001**			**<0.01**
Not present	**1.00**	**—**		**1.00**	**—**	
Present	**1.63**	**1.23, 2.17**		**1.70**	**1.17, 2.47**	
**Volume of metastases**			**0.03**			0.20
<20 metastases	**1.00**	**—**		1.00	—	
≥20 metastases	**1.50**	**1.07, 2.12**		1.31	0.85, 2.00	
**Metastatic lesion ≥5cm**			**0.01**			**<0.01**
Not present	**1.00**	**—**		**1.00**	**—**	
Present	**1.45**	**1.09, 1.93**		**1.74**	**1.21, 2.50**	
**Age**			0.17			0.06
<65 years	1.00	**—**		1.00	**—**	
≥65 years	1.01	1.00, 1.02		1.02	1.00, 1.03	
**Sex**			0.91			>0.9
Female	1.00	—		1.00	—	
Male	1.02	0.78, 1.32		0.99	0.71, 1.38	
**Smoking Status**			**0.02**			**0.02**
Never-Smoker	**1.00**	**—**		**1.00**	**—**	
Ex-Smoker	**0.58**	**0.40, 0.83**		**0.51**	**0.32, 0.82**	
Current Smoker	**0.62**	**0.41, 0.94**		**0.61**	**0.35, 1.06**	
**PD-L1 Status**			**<0.001**			**0.01**
<1%	**1.00**	**—**		**1.00**	**—**	
≥1%	**0.57**	**0.37, 0.88**		**0.74**	**0.44, 1.24**	
≥50%	**0.52**	**0.36, 0.74**		**0.63**	**0.41, 0.97**	
Not Tested	**0.95**	**0.66, 1.37**		**1.26**	**0.77, 2.07**	
**Tumour Type**			0.10			0.20
Adenocarcinoma	1.00	—		1.00	—	
SCC	1.56	1.14, 2.12		1.58	1.06, 2.34	

ULCC	1.23	0.80, 1.90		1.05	0.62, 1.78	
NOS	1.23	0.46, 3.34		0.63	0.15, 2.71	
**Treatment Type**			**<0.001**			**<0.001**
Chemotherapy	**1.00**	**—**		**1.00**	—	
Immunotherapy	**0.56**	**0.41, 0.78**		**0.52**	**0.31, 0.86**	
Chemoimmunotherapy	**0.54**	**0.40, 0.75**		**0.34**	**0.21, 0.56**	
**Radiotherapy**			0.64			0.80
No Upfront RT	1.00	—		1.00	—	
Upfront RT	1.07	0.80, 1.43		1.04	0.72, 1.50	

Legend: n: number; SCC: Squamous Cell Carcinoma; ULCC: Undifferentiated Large Cell Carcinoma; NOS: Not otherwise specified; PD-L1: Programmed Cell death ligand 1 receptor; RT: Radiotherapy

Supplementary Table S3: Univariate and Multivariate analysis for OS

	Univariate			Multivariate		
**Characteristic**	HR	95% CI	p-value	HR	95% CI	p-value
**Lung Metastases**			0.88			0.40
Not present	1.00	—		1.00	—	
Present	1.02	0.78, 1.34		1.15	0.81, 1.64	
**Lymph Node Metastases**			0.16			0.70
Not present	1.00	—		1.00	—	
Present	1.32	0.89, 1.95		1.09	0.69, 1.72	
**Liver Metastases**			0.90			0.09
Not present	1.00	—		1.00	—	
Present	1.02	0.73, 1.44		1.52	0.95, 2.43	
**Brain Metastases**			**<0.01**			>0.9
Not present	1.00	**—**		1.00	—	
Present	**1.64**	**1.15, 2.34**		1.00	0.62, 1.63	
**Adrenal Metastases**			0.61			0.30
Not present	1.00	—		1.00	—	
Present	0.91	0.63, 1.31		0.80	0.51, 1.24	
**Pleural Metastases**			0.94			0.40
Not present	1.00	—		1.00	—	
Present	1.01	0.76, 1.35		1.17	0.82, 1.68	
**Bone Metastases**			**<0.001**			**<0.001**
Not present	1.00	**—**		**1.00**	**—**	
Present	**1.95**	**1.45, 2.62**		**2.01**	**1.37, 2.97**	
**Volume of metastases**			**<0.01**			**0.03**
<20 metastases	1.00	**—**		**1.00**	**—**	
≥20 metastases	**1.77**	**1.24, 2.53**		**1.66**	**1.05, 2.62**	
**Metastatic lesion ≥5cm**			0.06			0.07
Not present	1.00	—		1.00	—	
Present	1.35	0.99, 1.83		1.44	0.98, 2.14	
**Age**			**0.02**			**<0.001**
<65 years	**1.00**	**—**		**1.00**	**—**	
≥65 years	**1.02**	**1.00, 1.03**		**1.03**	**1.01, 1.05**	
**Sex**			0.97			0.6
Female	1.00	—		1.00	—	
Male	1.00	0.76, 1.33		0.91	0.64, 1.28	
**Smoking Status**			0.13			0.20
Never-Smoker	1.00	—		1.00	—	
Ex-Smoker	0.68	0.46, 1.01		0.78	0.47, 1.28	
Current Smoker	0.84	0.54, 1.30		1.05	0.59, 1.87	
**PD-L1 Status**			0.06			0.40
<1%	1.00	—		1.00	—	
≥1%	0.74	0.48, 1.16		0.92	0.53, 1.60	
≥50%	0.58	0.40, 0.86		0.69	0.43, 1.10	
Not Tested	0.69	0.47, 1.01		0.71	0.41, 1.23	
**Tumour Type**			**0.02**			**<0.01**
Adenocarcinoma	**1.00**	**—**		**1.00**	**—**	
SCC	**1.76**	**1.29, 2.40**		**2.05**	**1.39, 3.04**	

ULCC	**0.96**	**0.58, 1.60**		**0.88**	**0.47, 1.63**	
NOS	**1.06**	**0.34, 3.33**		**0.36**	**0.05, 2.74**	
**Treatment Type**						
Chemotherapy	1.00		0.72	1.00		0.20
Immunotherapy	1.07	0.77, 1.48		1.65	0.98, 2.77	
Chemoimmunotherapy	1.16	0.82, 1.64		1.27	0.74, 2.17	
**Radiotherapy**						
No Upfront RT	1.00	—	0.43	1.00	—	0.70
Upfront RT	1.13	0.83, 1.54		1.07	0.73, 1.56	

Legend: n: number; SCC: Squamous Cell Carcinoma; ULCC: Undifferentiated Large Cell Carcinoma; NOS: Not otherwise specified; PD-L1: Programmed Cell death ligand 1 receptor; RT: Radiotherapy

Supplementary Table S4: OS and PFS for bone metastases by treatment type

Outcome	Immunotherapy				Chemoimmunotherapy				Chemotherapy			
	**Bone**	**No Bone**	**HR**	**p-value**	**Bone**	**No Bone**	**HR**	**p-value**	**Bone**	**No bone**	**HR**	**p-value**
**Median OS months (95% CI)**	5 (4-15)	21 (13-33)	**2.10**	**<0.01**	10 (8-21)	21 (16-NA)	**1.99**	**<0.01**	15 (9-35)	21 (16-30)	1.49	0.17
**1-year OS (%)**	32.0	60.6			40.7	68.5			56.2	67.2		
**2-year OS (%)**	16.0	43.3			21.5	43.8			25.0	47.7		
**Median PFS months (95% CI)**	2 (1-6)	9 (5-17)	**3.19**	**<0.001**	4 (4-9)	9 (7-NA)	**1.98**	**<0.01**	3 (1-10)	5 (4-5)	1.44	0.20
**6-month PFS (%)**	20.9	56.2			41.8	63.6			31.3	28		

Legend: HR: Hazard Ratio; CI: Confidence Interval; OS: Overall Survival; PFS: Progression Free Survival

Supplementary Table S5: OS and PFS for liver metastases by treatment type

Outcome	Immunotherapy				Chemoimmunotherapy				Chemotherapy			
	Liver	No Liver	HR	p-value	Liver	No Liver	HR	p-value	Liver	No Liver	HR	p-value
**Median OS months (95% CI)**	6 (2-NA)	16.5 (12-25)	**2.04**	**0.05**	9 (7-NA)	18 (14-29)	**1.80**	**0.05**	19.0 (12-35)	18.5 (14-30)	1.46	0.19
**1-year OS (%)**	30.0	55.8			33.4	63.2			66.7	64.7		
**2-year OS (%)**	10.0	39.3			0	35.1			40	44.1		
**Median PFS months (95% CI)**	1 (1-NA)	7 (5-12)	**3.19**	**<0.001**	4 (2-NA)	9 (6-13)	1.33	0.40	4 (3-5)	4 (3-9)	1.12	0.68
**6-month PFS (%)**	20.0	50.4			43.1	57.5			33.3	27.9		

Legend: HR: Hazard Ratio; CI: Confidence Interval; OS: Overall Survival; PFS: Progression Free Survival
